# Effects of low-level laser therapy on orthodontic miniscrew stability: a systematic review

**DOI:** 10.1186/s40001-023-01010-z

**Published:** 2023-01-27

**Authors:** De-Hua Zheng, Feng-Chun Hou, Yan-Jun Zang, Bing Li

**Affiliations:** 1 Department of Orthodontics, Qingdao Stomatological Hospital Affiliated to Qingdao University, Qingdao, Shandong People’s Republic of China; 2 Department of Genetics and Cell Biology, Basic Medical College, Qingdao University, Qingdao, China; 3 Department of Hematology, The Affiliated Hospital of Qingdao University, Qingdao, China; 4 No. 306, Ningxia Road, Shinan District, Qingdao, 266000 Shandong China

**Keywords:** Low-level laser therapy, Orthodontic, Miniscrews, Stability, Systematic review

## Abstract

**Background:**

Miniscrews as auxiliary anchorage devices in orthodontic treatment have definite advantages and efficacy. The aim of the present study was to investigate the scientific evidence including randomized controlled trials (RCTs) or controlled clinical trials (CCTs) to support the application of low-level laser therapy to improve miniscrews stability in orthodontic treatment.

**Methods:**

An extensive literature research was conducted with the Cochrane Library, PubMed, EMBASE, Web of Science and ScienceDirect without language limitations. All searches were inclusive until June 2020. The Cochrane Risk of Bias Tool was used to assess the risk of bias (RoB) in the included RCTs.

**Results:**

Through the electronic searches, 428 titles and abstracts were identified. From these, 4 articles were retrieved for review, and 3 of these met the inclusion criteria. Two RCTs reported increased miniscrews stability with low-intensity laser therapy, but the other one reported no difference. Except one study assessed as “high risk of bias” the other two were rated as “low risk of bias”.

**Conclusion:**

There is insufficient evidence to support or refute the effectiveness of LLLT for improvement of miniscrew stability. Further studies with a better study design, reliable evaluation method, comprehensive evaluation intervals and appropriate loading protocol are required to provide more reliable evidence for the clinical application of LLLT.

## Background

Anchorage control is one of the most important factors to be taken into account when planning optimal tooth movement in orthodontic treatment. Expectations are not always met, despite the applied different appliances, mechanics, elastics, wire bends and so forth [1]. Extraoral anchorage requires patients compliance and it is aesthetically unacceptable. The intramaxillary and intermaxillary anchorage load patients’ teeth, which may lead to their uncontrolled, mostly undesired movement [2]. The introduction of miniscrews system in contemporary orthodontics has been of great help in assisting complex treatment procedures such as maxillary molar distalization in nonextraction treatment [3], maxillary incisor retraction [4], intrusion of maxillary and mandibular molars [5] and correction of canted occlusal planes [6]. Since their introduction, orthodontic miniscrews have become very popular. Reasons might be because they have many advantages such as low price, removal, ease of insertion, rare complications and excellent anchorage control even in uncooperative patients [7]. However, one disadvantage of these anchorage devices is premature loss. The miniscrews stability may be one of the most important factors influencing its successful application in orthodontic treatment. Thus, it is fully justified that many studies focus on the relative factors influencing miniscrews stability. It is revealed that those factors may be oral hygiene, the quality of alveolar, the surgical protocol, the method of loading, the shape/size of miniscrews [8–10]. Accordingly, numerous strategies have been used to improve the stability of miniscrews and minimize the failure rate [11, 12].

Low-level laser therapy (LLLT) is a new internationally accepted designation and defined as laser treatment in which the energy output is low enough not to cause a rise in the temperature of the treated tissue above 36.5 °C or normal body temperature [13]. Recently, low-level laser therapy (LLLT) has attracted increasing attention because of its obvious advantages in analgesics, biostimulation, anti-inflammatory properties, regenerative effect and lack of adverse effects. The application of LLLT in orthodontics has shown to be effective in accelerating orthodontic tooth movement, preventing relapse and alleviating pain during orthodontic treatment [14–18]. The underlying mechanism might be multifaceted. Kawasaki et al. [19]. Reported that LLL irradiation-stimulated tooth movement was accompanied with an improvement on alveolar bone remodelling by increasing the number of osteoclasts, cellular proliferation of periodontal ligament cells, and mineralized bone formation. It has also been postulated that the beneficial effects of LLLT on pain relief can be attributed to modify nerve conduction by affecting the synthesis, release and metabolism of various neurochemicals, including endorphins and encephalin [20]. In addition to these beneficial effects for orthodontic treatment, LLLT has been reported to improve the stability of miniscrews and is suggested as a clinical adjuvant for increasing clinical success with miniscrew treatment [21]. However, Abohabib et al. [22] reported that LLLT cannot be an effective method to promote miniscrews stability. The effectiveness of laser in improving miniscrews stability in orthodontic treatment is therefore still uncertain.

Thus, a systematic review is essential for evidence-based clinical research and practice. The aim of the present study was to investigate the scientific evidence including randomized controlled trials (RCTs) or controlled clinical trials (CCTs) to support the application of low-level laser therapy to improve miniscrews stability in orthodontic treatment.

## Methods

The Preferred Reporting Items for Systematic Reviews and Meta-Analyses (PRISMA) checklist was used as a guideline for conducting this systematic review. The literature screening, data extraction and quality assessment were done independently by two authors. Any disagreements were resolved by discussion or by a third author.

### Eligibility criteria

Inclusion criteria are as follows:

1. Study design: the studies should be designed as randomized clinical trials (RCTs) or controlled clinical trials (CCTs), including split-mouth design.
2. Participants: patients received conventional fixed orthodontic appliance and required extraction of bilateral maxillary first premolars.
3. Intervention: low-level laser light was applied on the experimental side or experimental group.
4. Control: the placebo group/side received a pseudo-laser application in identical settings without laser activation. No laser treatment was conducted on the control group/side.
5. Outcome: the orthodontic miniscrew stability was monitored with resonance frequency analysis (RFA) or assessed using the periotest device.

Exclusion criteria were as follows:

1. Review articles, case reports, descriptive studies, animal experiments or laboratory studies.
2. The participants had any systematic diseases or taking any medications affecting gingival health and periodontal status. Other characteristics which have influence on the outcome.

### Search methods

An extensive literature research was conducted with the Cochrane Library, PubMed, EMBASE, Web of Science and ScienceDirect without language limitations. All searches were inclusive until November 2020. In addition, the reference lists of the retrieved articles were also reviewed by manual search. Keywords used in the search and combination of terms per database can be found in Table 1.

**Table 1:** Search strategy and results for Pubmed, EMBASE, Cochrane Library, Web of Science, and ScienceDirect

Database	Search strategy	Results
Pubmed, http://www.ncbi.nlm.nih.gov/pmc/	#1: Search (((((((((((((((((((((((((((((((Light Therapies, Low-Level) OR Light Therapy, Low-Level) OR Low Level Light Therapy) OR Low-Level Light Therapies) OR Therapies, Low-Level Light) OR Therapy, Low-Level Light) OR Photobiomodulation Therapy) OR Photobiomodulation Therapies) OR Therapies, Photobiomodulation) OR Therapy, Photobiomodulation) OR LLLT) OR Laser Therapy, Low-Level) OR Laser Therapies, Low-Level) OR Laser Therapy, Low Level) OR Low-Level Laser Therapies) OR Laser Irradiation, Low-Power) OR Irradiation, Low-Power Laser) OR Laser Irradiation, Low Power) OR Low-Power Laser Therapy) OR Low Power Laser Therapy) OR Laser Therapy, Low-Power) OR Laser Therapies, Low-Power) OR Laser Therapy, Low Power) OR Low-Power Laser Therapies) OR Low-Level Laser Therapy) OR Low Level Laser Therapy) OR Low-Power Laser Irradiation) OR Low Power Laser Irradiation) OR Laser Biostimulation) OR Biostimulation, Laser) OR Laser Phototherapy) OR Phototherapy, Laser #2: Search (((((((((((mini implants) OR mini-implants) OR screw implants) OR miniscrew implants) OR mini-screw implants) OR mini screw implants) OR microscrew implants) OR micro-screw implants) OR micro screw implants) OR microimplants) OR micro-implants) OR micro implants #3: Search Orthodontic* #4: #1 AND #2 AND #3	310,653 25,958 18,096 272
Embase, http://www.Embase.com/	#1. ‘low level laser therapy’:ab,ti #2. ‘endoscopic laser therapy’:ab,ti #3. ‘laser biostimulation’:ab,ti #4. ‘laser therapy, low-level’:ab,ti #5. ‘low energy laser therapy’:ab,ti #6. ‘low energy laser treatment’:ab,ti #7. ‘low intensity laser therapy’:ab,ti #8. ‘low intensity laser treatment’:ab,ti #9. ‘low level laser treatment’:ab,ti #10. ‘low level light therapy’:ab,ti #11. ‘low power laser therapy’:ab,ti #12. ‘low power laser treatment’:ab,ti #13. ‘low-level light therapy’:ab,ti #14. ‘miniscrew’:ab,ti #15. ‘mini implants’:ab,ti #16. ‘screw implants’:ab,ti #17. ‘miniscrew implants’:ab,ti #18. ‘microscrew implants’:ab,ti #19. ‘microimplants’:ab,ti #20. ‘micro implants’:ab,ti #21. Orthodontic #22. ‘low level laser therapy’/exp #23. #1 OR #2 OR #3 OR #4 OR #5 OR #6 OR #7 OR #8 OR#9 OR #10 OR #11 OR #12 OR #13OR #22 #24. ‘miniscrew’/exp #25. #14 OR #15 OR #16 OR #17 OR #18 OR #19 OR #20 OR #24 #26. ‘randomized controlled trial’/exp #27. #21 AND #23 AND #25 AND #26	2388 203 98 7 49 19 168 18 91 161 71 24 161 530 601 322 107 19 159 107 44,184 20,137 20,745 16 1698 526,646 0
Cochrane Library, http://cochranelibrary.com/	#1: MeSH descriptor: [Low-Level Light Therapy] explode all trees #2: (“low level laser therapy”):ti,ab,kw (Word variations have been searched) #3:(Laser Biostimulation):ti,ab,kw (Word variations have been searched) #4: (Photobiomodulation Therapy):ti,ab,kw (Word variations have been searched) #5: (Low-Power Laser Irradiation):ti,ab,kw (Word variations have been searched) #6: #1 or #2 or #3 or #4 or #5 #7: (miniscrew):ti,ab,kw (Word variations have been searched) #8: (mini-implant):ti,ab,kw (Word variations have been searched) #9: (micro-implant):ti,ab,kw (Word variations have been searched) #10: (orthodontic implant):ti,ab,kw (Word variations have been searched) #11: MeSH descriptor: [Orthodontics] explode all trees #12: #7 or #8 or #9 or #10 #13: #6 AND #11 AND #12	814 1550 46 138 46 2070 62 67 12 118 2534 192 1
Web of science, http://apps.webofknowledge.com/	#1 (((((((((((((((((((((((((((((((Light Therapies, Low-Level) OR Light Therapy, Low-Level) OR Low Level Light Therapy) OR Low-Level Light Therapies) OR Therapies, Low-Level Light) OR Therapy, Low-Level Light) OR Photobiomodulation Therapy) OR Photobiomodulation Therapies) OR Therapies, Photobiomodulation) OR Therapy, Photobiomodulation) OR LLLT) OR Laser Therapy, Low-Level) OR Laser Therapies, Low-Level) OR Laser Therapy, Low Level) OR Low-Level Laser Therapies) OR Laser Irradiation, Low-Power) OR Irradiation, Low-Power Laser) OR Laser Irradiation, Low Power) OR Low-Power Laser Therapy) OR Low Power Laser Therapy) OR Laser Therapy, Low-Power) OR Laser Therapies, Low-Power) OR Laser Therapy, Low Power) OR Low-Power Laser Therapies) OR Low-Level Laser Therapy) OR Low Level Laser Therapy) OR Low-Power Laser Irradiation) OR Low Power Laser Irradiation) OR Laser Biostimulation) OR Biostimulation, Laser) OR Laser Phototherapy) OR Phototherapy, Laser #2: (((((((((((mini implants) OR mini-implants) OR screw implants) OR miniscrew implants) OR mini-screw implants) OR mini screw implants) OR microscrew implants) OR micro-screw implants) OR micro screw implants) OR microimplants) OR micro-implants) OR micro implants #3: Orthodontic* #4: #1 AND #2 AND #3	10,951 19,911 24,056 9
Science Direct, https://www.sciencedirect.com/	Title, abstract, or keywords: (“mini-implants” OR “miniscrew implants” OR “microscrew implants”)AND(“low level laser therapy” OR “Photobiomodulation Therapy” OR “Laser Biostimulation” OR “low intensity laser therapy” OR “low level light therapy’)	290

### Study selection

The relevant articles were selected through a two-phase process. In phase 1, two authors (DH Zheng and FC Hou) systematically analysed the titles and abstracts independently. The articles in which titles met the objectives of the study were selected for phase 2. In the second phase, full text were obtained for preliminarily eligible studies, and these were evaluated to verify whether they fulfilled the eligibility criteria. Any disagreement was settled by means of discussion until a mutual consensus was reached. Finally, three studies were included in this review: Osman et al. [21] Ahmed Mohamed Abohabib et al. [22] Abdullah Ekizer et al. [23]

### Risk of bias in individual studies

The Cochrane Risk of Bias Tool [24] was used to assess the risk of bias (RoB) in the included RCTs. An overall unclear/high RoB was given to the study when at least one domain from the seven domains was judged as unclear/high RoB.

### Synthesis of studies

The following data were extracted from each included study: author and year, age, sample size, study design, implant dimension, implant sites, evaluation methods, evaluation interval, statistical analysis, conclusion, type of laser, wavelength, output energy, methods of application, frequency of laser treatment. As a result of available data, a meta-analysis was not possible. Included studies assessed miniscrew stability by different devices. In the study of Osman et al. [21]. Minscrew stability was clinically assessed using the periotest device (Siemens AG, Bensheim, Germany). However, in other studies (Ahmed Mohamed Abohabib et al. [22] and Abdullah Ekizer et al. [23]), miniscrew stability was monitored with resonance frequency analysis (RFA) using the Osstell ISQ device (Osstell, Gothenburg, Sweden). In addition, in order to fit the AbsoAnchor miniscrew, a modified SmartPeg type 1 and resonance frequencies values in hertz were used in the study of Ahmed Mohamed Abohabib et al. [22]. Resonance frequencies values in hertz could not be compared to other studies which produced implant stability quotient (ISQ) value by resonance frequency analysis (RFA) due to the nature of the modification.

## Results

### Study selection

**Fig. 1:**
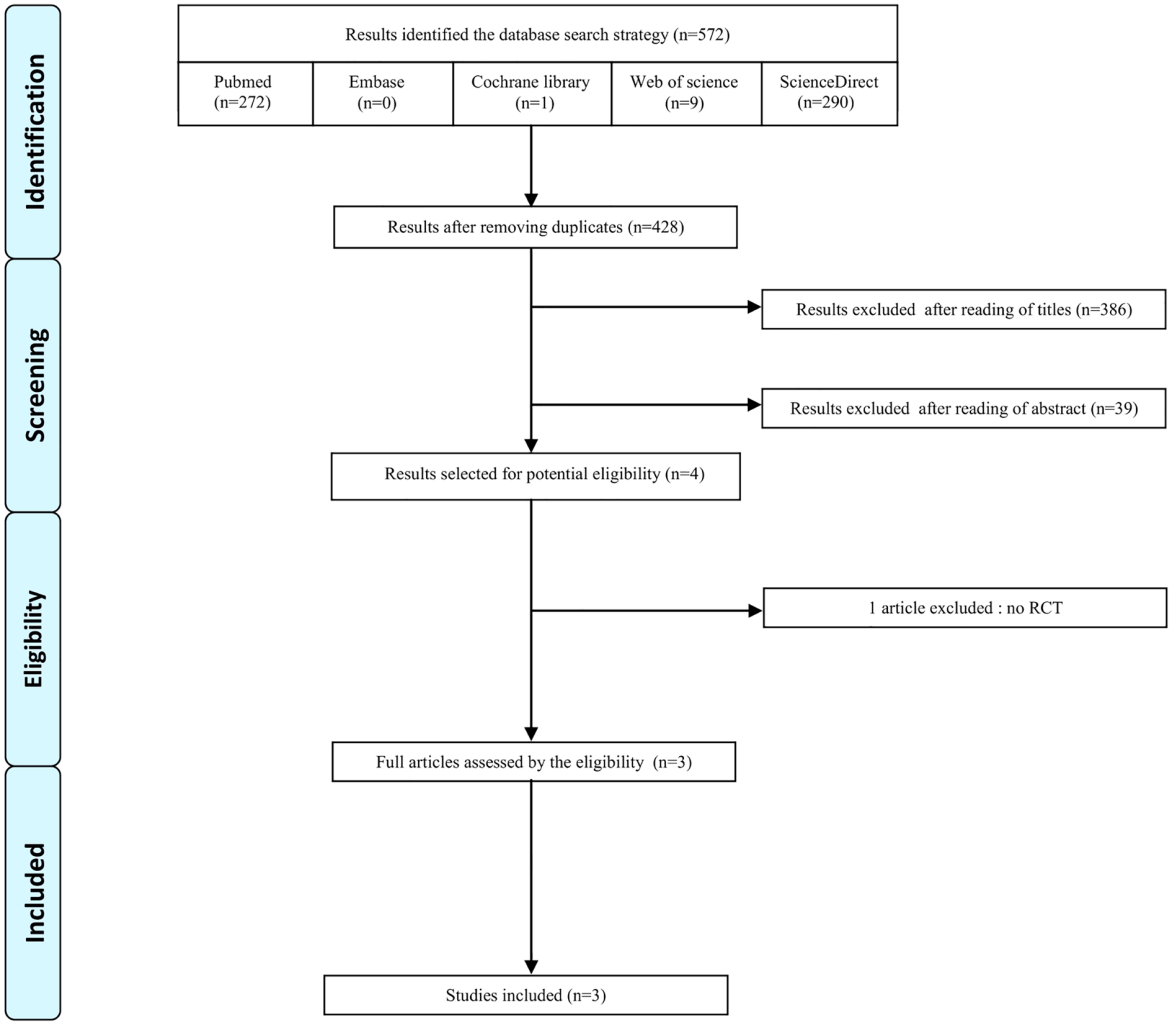
PRISMA flow diagram of the study inclusion process

The flow diagram (Fig. 1) describes the results of search queries. The search retrieved 372 articles from Pubmed, 1 from Cochrane library, 9 from Web of science, 290 from Science Direct, and none from Embase. After removal of duplicate citations, a total 428 articles were screened by reading titles and abstracts, and 424 studies were excluded. After screening the full-text articles of the remaining 4 studies, a total of 3 eligible studies were included for the systematic review.

### Study characteristics

Table 2 shows a summary of the characteristics about the three included studies. All included studies placed the mini-implant on the buccal region between the roots of maxillary second premolar and first molar. Three studies encompassing 46 subjects. All of them used a split-mouth RCT design. Their radiation was performed on the test side of maxilla (miniscrew insertion area), whereas the other side was chosen the control without irradiation but applied similar device as a placebo.

**Table 2:** Characteristics of included studies

Author and year	Age (years)	Sample size(M/F)	Design	Miniscrew dimension (diameter/length in mm)	Implanted sites	Evaluation method	Evaluation interval	Statistical analysis	Study conclusion
Abdullah Ekizer et al. (2016)	16.77 ± 1.4 (19–13.1)	20 (7/13)	Split-mouth RCT	1.6/8	Buccal region between the roots of maxillary second premolar and first molar	Resonance frequency analysis (RFA) Osstell ISQ device (Osstell, Gothenburg, Sweden)	Miniscrew placement (baseline), 1, 2 and 3 months	Paired samples *t*-test were used at *P* < 0.05 level	Photobiomodulation therapy had positive effect on stability of miniscrews during canine distalization
Osman A et al. (2017)	18 (14–32)	12 (6/6)	Split-mouth RCT	1.5/8	Buccal region between the roots of maxillary second premolar and first molar	Damping capacity assessment (DCA) Periotest device (Siemens AG, Bensheim, Germany)	Before and after loading 7, 14, 21, 30 and 60 days	Paired samples *t*-test were used at *P* < 0.05 level	LLLT can be suggested as a clinical adjuvant for improving clinical success with miniscrew treatment. However, more long-term studies incorporating more study subjects is deemed necessary to further validate the study results
AhmedMohamed Abohabib et al. (2018)	20.9 ± 3.4 (16–25)	14	Split-mouth RCT	1.5/8	Buccal region between the roots of maxillary second premolar and first molar	Resonance frequency analysis (RFA) Osstell device (Osstell, Gothenburg, Sweden)	0,1,2,3,4,6,8,10 weeks after mini-implant placement	Paired samples *t*-test was used at *P* < 0.05 level to compare two groups, Tukey’s post hoc test was used to study the changes of time in each group	Despite evidence of some significant differences in resonance frequency between mini-implants exposed to low-intensity laser light over a 10-week period there were no differences in mini-implant stability. LLLT cannot be recommended as a clinically useful adjunct to promoting mini-implant stability during canine retraction

The details of the lasers and parameters are shown in Table 3. Osman et al. [21] and Ahmed Mohamed Abohabib et al. [22] used a Biolase diode laser, with a wavelength between 910 and 940 nm. However, Abdullah Ekizer et al. [23] used OsseoPulseLED device with a wavelength of 618 nm. Although two of included studies [22, 23] used resonance frequency analysis (RFA) to evaluate the miniscrews stability, one study used Osstell ISQ RFA device to produce an implant stability quotient (ISQ) index, the other used a modified SmartPeg of Osstell RFA device to produce the average values of two perpendicular directions in hertz. The study of Osman et al. [21] used damping capacity assessment (DCA) conducted by periotest device to assess miniscrew stability.

**Table 3:** The parameters and regimen of diode laser applied in included studies

Study ID	Type of laser	Wavelength	Output/energy (density)	Method of application	Frequency of laser treatment
Abdullah Ekizer et al.	OsseoPulseLED device (BioluxResearch Ltd, Vancouver,Canada)	618 nm	20 mW/cm^2^	The LED irradiation was performed on the test side of maxilla, whereas the other side was chosen the control without irradiation but applied similar device	20 min once a day during 21 days
Osman A et al.	Diode laser (Biolase Technology, Inc.; San Clemente, Calif, USA)	910 nm	0.7 watts for 60 s	The irradiation was applied over the mini screw insertion area. On the contralateral side, the bio stimulation tip of the laser was directed toward the miniscrew while the laser device was switched off to act as a placebo	The irradiation was repeated throughout the duration of 14 days with an interval of 72 h between each application (four applications)
AhmedMohamed Abohabib et al.	Biolase (Epic 10 Console) diode laser	940 nm	36 J/cm^2^	The laser was placed over and perpendicular to the mini-implant on an active or working mode for the laser sides; whilst on the control sides, the laser was placed over the mini-implant without using the active mode	LLLT was applied on days (0, 7, 14 and 21) after mini-implant placement

With regard to the evaluation interval, Abdullah Ekizer et al. [23] recorded three consecutive times at T0 (miniscrew loading), T1 (1 month after placement), T2 (2 months after placement) and T3 (3 months after placement). Osman et al. [21] assessed the miniscrew stability at different time intervals, immediately after placement, after days 7, 14, 21, 30, 60. However, Ahmed Mohamed Abohabib et al. [22] measured miniscrew stability using resonance frequency analysis at 0, 1, 2, 3, 4, 6, 8, 10 weeks after miniscrew placement.

### Risk of bias (RoB) within the included studies

**Fig. 2:**
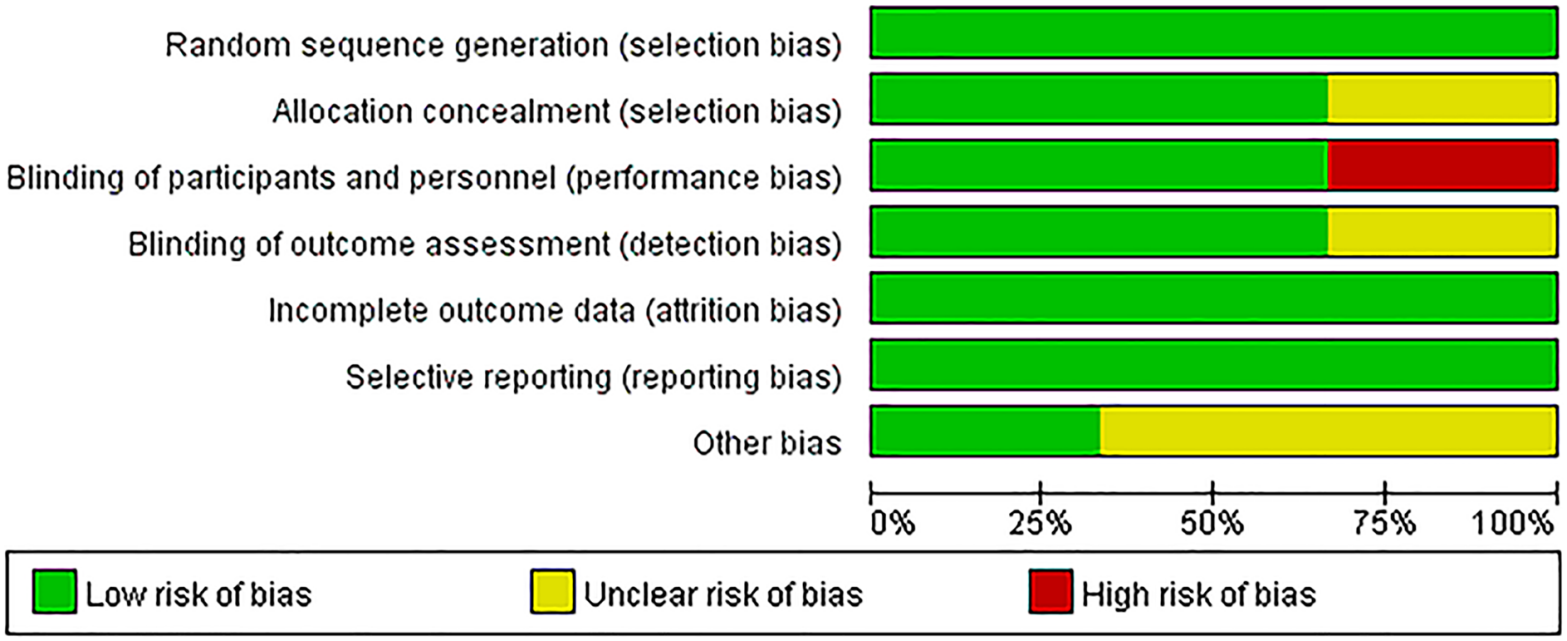
Risk of bias graph: review authors’ judgements about each risk of bias item presented as percentages across all included studies

**Fig. 3:**
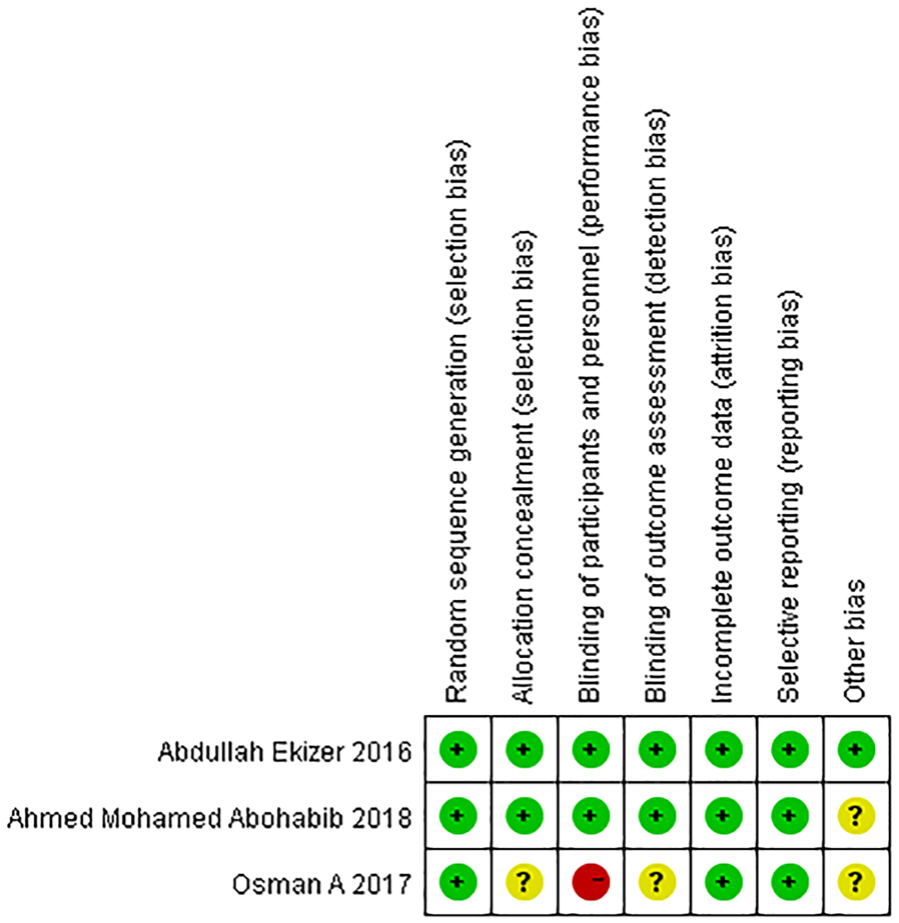
Risk of bias summary: review authors’ judgements about each risk of bias item for each included study

The details of RoB assessment are summarized in Figs. 2, 3 and Table 4.

**Table 4:** Assessment of risk of bias

Article	Judgement	Support for judgement
Abdullah Ekizer et al. 2016		
Random sequence generation (selection bias)	Low risk	Quote: Trial and placebo sides were selected on a random basis. (coin toss)
Allocation concealment (selection bias)	Low risk	Quote: Twenty envelopes containing the subjects’ information were used to ensure allocation concealment from the researchers. Sequential numbers were written on the envelopes
Blinding of participants and personnel	Low risk	Both the investigators and the participants remained blinded to treatment
Blinding of outcome assessment (detection bias)	Low risk	Quote: The measurements of the data were done by a clinician blinded to the assignment
Incomplete outcome data (attrition bias)	Low risk	All randomized patients were accounted for
Selective reporting (reporting bias)	Low risk	No suggestion of incomplete reporting of data and outcome
Other bias	Low risk	Study appears to be free of other sources of bias
Osman A et al. 2017		
Random sequence generation (selection bias)	Low risk	Quote: Both sides were randomly selected by tossing a coin, in which the face was the study side and the back was the control side for each selected patient
Allocation concealment (selection bias)	Unclear risk	Method of concealment was not mentioned
Blinding of participants and personnel	High risk	Participants was blind, but not Clinician and investigator
Blinding of outcome assessment (detection bias)	Unclear risk	it was not mentioned whether blinding of outcome assessors was done or not
Incomplete outcome data (attrition bias)	Low risk	All randomized patients were accounted for
Selective reporting (reporting bias)	Low risk	No suggestion of incomplete reporting of data and outcome
Other bias	Unclear risk	Source of funding was not mentioned
Ahmed Mohamed Abohabib et al. 2018		
Random sequence generation (selection bias)	Low risk	Quote: Block randomization was performed with a 1:1 allocation. The sequence of the sides subjected to low-intensity laser light and the control was computer-generated with random numbers
Allocation concealment (selection bias)	Low risk	Quote: The allocation was carried out using opaque sealed envelopes. On the day of mini-implant placement, which was also the first day of laser light application, each subject chose one envelope to detect their randomized allocation
Blinding of participants and personnel	Low risk	Both the clinician and the participants remained blinded to treatment
Blinding of outcome assessment (detection bias)	Low risk	Assessor measured the stability values blindly for each side at every evaluation interval
Incomplete outcome data (attrition bias)	Low risk	Dropout patients were reported and the reasons were mentioned
Selective reporting (reporting bias)	High risk	No suggestion of incomplete reporting of data and outcome
Other bias	Unclear risk	Source of funding was not mentioned

### Effect of interventions

Because of the heterogeneity in the measuring devices and the methods of outcomes measurement, it was impossible to combine the data for meta-analysis. Osman et al. [21] reported a non-statistically significant difference between experimental and control side values, while the mean mobility measures in the experimental sides was less than the control sides during the whole of the observation period (7, 14, 21, 30 and 60 days). Ahmed Mohamed Abohabib et al. [22] reported some minor favourable changes in resonance frequency scores at 3, 4, 6, 8 weeks after mini-implant loading using low-intensity laser therapy although clinical stability was not affected. These changes are less than some reported in previous studies [25, 26]. When comparing changes between low laser therapy and control sides, no statistical difference of ISQ values was found at 1st month in the study of Abdullah Ekizer et al. [23]. However, miniscrew stability was significantly different for low laser therapy and control sides at 2nd month and 3rd month.

## Discussion

Miniscrews as auxiliary anchorage devices in orthodontic treatment have definite advantages and efficacy. The successful of miniscrews in providing definitive anchorage depends on its stability. Improvement of miniscrew stability has great importance to achieving successful skeletal anchorage and successful treatment outcome. Low-level laser therapy (LLLT) is an attractive noninvasive option with many benefits including stimulation/inhibition of physiological, biochemical or proliferative activities which are associated with accelerating the osseointegration of miniscrews. Therefore, a systematic review assessing the clinical evidence has Qhigh importance.

Osman et al. used Periotest device (periotest values, PTVs) along with damping capacity analysis (DCA) to evaluate the stability of the miniscrew implant, which although DCA system is user-friendly and time- and cost-efficient, it is also been reported that this method can be affected by a variety of factors such as implant location and angulation, positioning of device handpiece (horizontal distance and angle of the implant). These factors may result in low reproducibility and sensitivity [27–30]. Abdullah Ekizer et al and AhmedMohamed Abohabib et al used Osstell Device ISQ along with resonance frequency analysis (RFA) to measure the stability of the miniscrew implant. Resonance frequency analysis is a noninvasive measurement technique that holds great promise for the clinical evaluation of mini-implant stability. The Osstell device utilizes the basic principles of the harmonic response method. However, the Osstell is used by screwing the transducer into the dental implant with a torque of 10 Ncm, which is almost half the force used for implant insertion and may result in microdamage [31]. Moreover, when the device is used for RF analysis, stable coupling between the SmartPeg transducer and the implant is necessary. However, a transducer (SmartPeg) suitable for the size and structure of particular miniscrews and modified miniscrews fit the Osstell transducer may be difficult to obtain [32, 33]. In addition, there were no standard values for the success or failures of the orthodontic miniscrew, so failure had to be assessed clinically as Osstell devise has calibrated values only for dental implants which could not be applied for orthodontics. Although both methods are used to measure implant stability, correlation and reliability between ISQs and PTVs is still a controversial issue. A recent systematic analysis found that there is no consensus and standardization in the assessment of implant stability related to the values obtained by RFA and DCA devices, which could create disagreements and miscommunication among dentistry among professionals [34]. Therefore, a preferred method or modified device which could minimize contact, avoid torque, disassembly and discrepancies in evaluation criteria is needed to assess the stability of orthodontic miniscrew specially.

After miniscrew placement, mechanical stability is gradually replaced by biological secondary stability. A critical interval in the healing process is the period during which osteoclastic activity has decreased the initial mechanical stability of the implant but new bone formation is still insufficient to maintain implant stability. Ure et al. [31] who reported that the stability of mini-implant decreased during the first three weeks and increased thereafter. Carney et al. [35] reported that a decrease in stability was noted during the first three weeks, while an increase in stability over the next two weeks was revealed, then followed by a decrease in stability after the fifth week. The study of AhmedMohamed Abohabib et al. containing comprehensive and continuous observation time (baseline to 10 weeks), finding that from baseline to week 1, the sides expose to the low-intensity laser showed no significant changes in mean resonance frequency values, from week 1 to 2, there was a significant decrease in mean resonance frequency values, from week 3 to week 10, the low-intensity laser sides showed significantly increased mean resonance frequency values compared to control sides. However, Abdullah Ekizer et al. recorded three consecutive times at baseline, 1 month, 2 months after 3 months after placement. These evaluation intervals may ignore the effect of low-intensity laser on the transition from mechanical stability to biological stability in healing process.

There are multiple loading protocols of miniscrew reported in the literature. The timing of the loading recommended in the literature ranges from immediate to 3 months post-operatively, although most of the authors deemed immediate loading possible and rational, provided a low force value is applied [36]. Nevertheless, it seems reasonable to postpone loading for 2 weeks after micro-implant placement, in order to allow uneventful healing of the mucosa around the miniscrew heads, which is crucial to prevent inflammation: one of the major causes of micro-screw failures [37]. According to Osman et al. and Abdullah Ekizer et al. both the experimental sides and control sides were loaded with a force of 150 g for canine retraction after 14 days of miniscrew insertion. However, in the study of AhmedMohamed Abohabib et al. miniscrew were immediately loaded with a force of 150 g with split-mouth randomization to low-intensity laser-treated side and control side. Therefore, it is still uncertain whether different loading protocols resulted in discrepancies of stability of miniscrew among included studies. It is suggested that when assessing the effect of low intensity laser therapy on miniscrew stability, postponement of loading time should be used in all analysis.

According to assessment of risk of bias, only one study had low risk of bias (Abdullah Ekizer et al.). One study presented an unclear risk (Ahmed Mohamed Abohabib et al.). The study of Osman et al. was judged to have a high risk of bias, since clinician and investigator participated in this study was not blinded. It is possible that this may have had effect on their PTVs and ISQs. Additionally, method of concealment and blinding of outcome assessor were not mentioned in their report. Therefore, “Allocation concealment”, “Blinding of participants and personnel” and “Blinding of outcome assessment” were the main weaknesses in their study. The high or unclear risk of bias means that the results must be interpreted with caution.

## Conclusions

Because of extensive methodological weakness and significant heterogeneity of the existing evidence, there is insufficient evidence to support or refute the effectiveness of LLLT for improvement of miniscrew stability. Further studies with a better study design, reliable evaluation method, comprehensive evaluation intervals and appropriate loading protocol are required to provide more reliable evidence for the clinical application of LLLT.

## Data Availability

All information provided in this article is available from the corresponding author on reasonable request.
